# Deficiency of leukocyte-specific protein 1 (LSP1) alleviates asthmatic inflammation in a mouse model

**DOI:** 10.1186/s12931-022-02078-7

**Published:** 2022-06-22

**Authors:** Nguyen Phuong Khanh Le, Amanda Florentina do Nascimento, David Schneberger, Chi Cuong Quach, Xiaobei Zhang, Gurpreet K. Aulakh, Wojciech Dawicki, Lixin Liu, John R. Gordon, Baljit Singh

**Affiliations:** 1 Veterinary Biomedical Sciences, University of Saskatchewan, Saskatoon, Canada; 2 Faculty of Animal Science and Veterinary Medicine, Nong Lam University, Ho Chi Minh City, Vietnam; 3 Small Animal Clinical Sciences, University of Saskatchewan, Saskatoon, Canada; 4 Department of Medicine, University of Saskatchewan, Saskatoon, Canada; 5 Department of Anatomy, Physiology and Pharmacology, University of Saskatchewan, Saskatoon, Canada; 6 Western College of Veterinary Medicine, University of Saskatchewan, Saskatoon, S7N5B4 Canada

**Keywords:** Macrophages, Leukocytes, LSP1, Asthma model

## Abstract

**Background:**

Asthma is a major cause of morbidity and mortality in humans. The mechanisms of asthma are still not fully understood. Leukocyte-specific protein-1 (LSP-1) regulates neutrophil migration during acute lung inflammation. However, its role in asthma remains unknown.

**Methods:**

An OVA-induced mouse asthma model in LSP1-deficient (Lsp1^−/−^) and wild-type (WT) 129/SvJ mice were used to test the hypothesis that the absence of LSP1 would inhibit airway hyperresponsiveness and lung inflammation.

**Results:**

Light and electron microscopic immunocytochemistry and Western blotting showed that, compared with normal healthy lungs, the levels of LSP1 were increased in lungs of OVA-asthmatic mice. Compared to Lsp1^−/−^ OVA mice, WT OVA mice had higher levels of leukocytes in broncho-alveolar lavage fluid and in the lung tissues (P < 0.05). The levels of OVA-specific IgE but not IgA and IgG1 in the serum of WT OVA mice was higher than that of Lsp1^−/−^ OVA mice (P < 0.05). Deficiency of LSP1 significantly reduced the levels of IL-4, IL-5, IL-6, IL-13, and CXCL1 (P < 0.05) but not total proteins in broncho-alveolar lavage fluid in asthmatic mice. The airway hyper-responsiveness to methacholine in Lsp1^−/−^ OVA mice was improved compared to WT OVA mice (P < 0.05). Histology revealed more inflammation (inflammatory cells, and airway and blood vessel wall thickening) in the lungs of WT OVA mice than in those of Lsp1^−/−^ OVA mice. Finally, immunohistology showed localization of LSP1 protein in normal and asthmatic human lungs especially associated with the vascular endothelium and neutrophils.

**Conclusion:**

These data show that LSP1 deficiency reduces airway hyper-responsiveness and lung inflammation, including leukocyte recruitment and cytokine expression, in a mouse model of asthma.

**Supplementary Information:**

The online version contains supplementary material available at 10.1186/s12931-022-02078-7.

## Background

As one of the most prevalent chronic respiratory diseases, asthma is responsible for huge economic losses and high mortality [1]. The pathogenesis of this disease is complicated because of the combination of genetic and environmental factors [2, 3]. Consensus statements regarding the various phenotypes and endotypes of asthma have been developed by ATS/ERS [4]. Asthmatic patients have symptoms such as chest tightness, shortness of breath, wheezing, and coughing, especially early in the morning or during the night. The clinical signs during an asthma attack are an outcome of increased amounts of mucous in the airways, narrowing of the airway lumen, contraction of hypertrophied smooth muscles and inflammation. The most commonly used mouse model of ovalbumin (OVA)-induced asthma mimics acute asthma [5].

Several changes are observed in the airways of asthmatic lungs. Firstly, there is exuberant migration of inflammatory cells including T lymphocytes, eosinophils, macrophages, and neutrophils into the lung. Secondly, airway albumin levels are significantly increased across the spectrum of asthma severity and are correlated with tryptase levels [6–9]. Thirdly, the airway epithelium suffers pathologic changes characterized by shedding of ciliated columnar cells and goblet and squamous cell metaplasia. The sub-epithelial basement membrane thickens due to fibroblast activation and deposition of extracellular matrix (e.g., collagen) [10]. Fourthly, mucus plugging is a common feature in the case of acute asthma [11]. The main reason for high mortality in asthmatic patients is airway obstruction due to airway hyperresponsiveness (AHR) and mucus hypersecretion by goblet cells, causing asphyxia [12, 13].

There is a clinical and pathologic correlation between the eosinophilic and neutrophilic inflammation and the severity of asthma [11]. The tracheal mucus aspirated from acute severe asthmatic humans has more aggregated neutrophils than eosinophils, consistent with increased levels of the neutrophil chemoattractant CXCL8/IL-8 [14]. In vitro data suggests that human neutrophil elastase enhances eosinophil degranulation and eosinophil cationic protein production [15]. In chronic asthma, sputum eosinophil percentages are strongly associated with reduced forced expiratory volume (FEV1) values. Similarly, sputum neutrophil percentages are positively correlated with older age and lower levels of the pre-bronchodilator FEV1 [16, 17]. Thus, neutrophilic airway inflammation is thought to play a major role in the progression of persistent airflow limitation in asthma [16]. It is interesting that most asthma treatments neither control neutrophil migration in severe cases [17] nor hasten clearance of neutrophils [18]. The lack of effective treatment for the 5–10% of cases that comprise severe asthma account for bulk of the asthma-related healthcare costs [19]. Inflammatory mediators such as IL-4, IL-13 and CXCL1 have important regulatory roles in asthmatic cell recruitment and activation [20, 21]. It appears that excessive migration of neutrophils and eosinophils may underlie the inflammation-associated structural and physiologic changes in the asthmatic lung. Therefore, a better understanding of molecular regulation of their migration may provide better ways of managing asthma.

Leukocyte-specific protein 1 (LSP1), discovered in 1988 in lymphocytes [22, 23] and initially named lymphocyte-specific protein 1, is now found in monocytes, macrophages, neutrophils, and endothelium [22–27]. The varied functions of LSP1, in various organs and in distinct contexts, are still complicated and poorly understood. This protein plays an important role in leukocyte chemotaxis in inflamed organs [28]. We reported that the absence of LSP1 moderated endotoxin-induced acute lung inflammation in a mouse model and reduced migration of neutrophils into the lungs [29]. Although there were no differences in MAPK phosphorylation between endotoxin challenged Lsp1-/- and WT mice, our data pointed towards a direct role of phosphorylated LSP-1 in modulating neutrophil cytoskeleton [29]. Increased expression of LSP1 has been implicated in Neutrophil Actin Dysfunction disorder, which is a rare immunologic condition [30], but LSP1 has also been implicated in T-cell migration in rheumatoid arthritis [31]. Because we still don’t fully understand the mechanisms that regulate migration of neutrophils and eosinophils in asthma, we tested a hypothesis that, in a mouse model of OVA-induced asthma, deficiency of LSP1 will suppress airway inflammation by inhibiting inflammatory cell recruitment into the lungs. We found that Lsp1^−/−^ asthmatic mice showed significantly decreased inflammatory cell emigration into the lungs and lower levels of related cytokines in broncho-alveolar lavage (BAL) fluids, as well as serum IgE, airway hyperresponsiveness (AHR), and histopathology.

## Methods

### Murine asthma model and airway hyperresponsiveness (AHR) measurement

LSP1-deficient (Lsp1^−/−^) mice were generated by Dr. Jenny Jongstra-Bilen and colleagues on the background of 129/SvJ mice at the University of Toronto [32, 33]. Both WT and the LSP knockout strains were transferred to and bred in the Laboratory Animal Services Unit at the University of Saskatchewan. The mice used in this study were produced just after the backcrossing and genotyping of Lsp1^−/−^. Sixteen-week-old male wild-type (WT) 129/SvJ and Lsp1^−/−^ mice were used (n = 6 mice per treatment group). All the animal experiments were approved by the University of Saskatchewan’s Animal Research Ethics Board and adhered to the Canadian Council on Animal Care guidelines. All mice were housed in a 12-h dark/light cycle, were fed a standard laboratory diet in the Laboratory Animal Services Unit at the University of Saskatchewan and allowed to acclimatize for one week before treatment. The OVA-induced asthma mouse model was designed as described previously [34, 35]. In general, mice were injected intraperitoneally (i.p.) with 2 µg OVA/2 mg alum twice, two weeks apart; two weeks they were given three aerosol challenges with 1% OVA in saline for 20 min per day, 2 days apart. Two weeks after the final aerosol challenge, AHR to methacholine (MCh) was determined using a head-out plethysmograph and a small animal ventilator (Kent Scientific, Litchfield, CT) and changes in the airflow were monitored with a flow sensor (TRS3300; Kent Scientific) linked via a preamplifier and A/D board (Kent Scientific) to a computer-driven real-time data acquisition/analysis system (DasyLab 5.5; DasyTec USA, Amherst, NH). [35, 36]. AHR data reflects 50% point in the expiratory cycle (Flow@50%TVe1) responding to the aerosols of saline 0.9%, then doubling doses of MCh (1.5–25 mg/mL) which enhance airway contraction [37, 38]. The next day all mice were challenged with 1% OVA in saline aerosols at a delivery rate of 0.5 L/min in an enclosed flow-through chamber for 20 min using an ultrasonic nebulizer (Ultra-Neb 99 by Devilbiss, Somerset, PA). After 24 h, the mice were euthanized with 200 mg/kg ketamine hydrochloride (Vetalar® injection U.S.P, Bioniche, Belleville, ON, Canada) and 10 mg/kg xylazine (Rompun®, Bayer, Toronto, ON, Canada) followed by collection of blood, BAL fluids and lung tissues.

### Blood and broncho-alveolar lavage cell counts

BAL fluid was collected as described previously [29]. Briefly, the trachea was exposed and the airways were lavaged with 1.5 mL of cold sterile 0.1 M PBS supplemented with 0.01% bovine serum albumin. The BAL fluid was centrifuged at 1500*g* for 10 min at 4 °C and the supernatants collected and stored at − 80 °C for protein, chemokine, and cytokine detection. The BAL fluid total leukocytes were counted and the cells resuspended at 106 cells/ml in 0.1 M PBS. One hundred µL of each sample was cytospun onto a microscope slide, and the cells were stained with Hemacolor stain kit (EMD Chemicals, Gibbstown, NJ, USA) for differential leukocyte counts (4 random fields at 400 × magnification).

Peripheral blood was collected into heparinized tubes by cardiac puncture. The total number of leukocytes/ml of blood was assessed after erythrocyte hemolysis with 2% acetic acid [39]. Simultaneously, a blood smear was stained with Hemacolor stain kit for differential leukocyte count in 10 fields at 400 × magnification.

### Cytokine and chemokine analyses in BAL fluid

BAL fluid levels of interleukin 4 (IL-4), IL-5, IL-6, IL-13, IL-17, interferon-γ (IFN-γ), CCL11 (eotaxin-1) and CXCL1 (keratinocyte-derived chemokine) were quantified using Bio-Plex Pro assays kit (Bio-Rad, Mississauga, ON, Canada), following the manufacturer’s instructions. Briefly, 96-well plates were washed with Bio-Plex assay buffer before multiplex bead working solution was added. Then beads were washed, after which diluted standards and samples were added to the wells. Detection antibodies were next added, followed by 1 × streptavidin-PE. Washing unbound proteins with Bio-Plex wash buffer was done in between each step. The plate was read on the Bio-Plex system (Bioplex 200 Luminex machine with Bioplex manager 6.1 software).

### Enzyme-linked immunosorbent assay (ELISA) measuring OVA-specific IgA, IgG1, IgE

ELISA was used to detect OVA-specific antibody IgA, IgG1 [35] and IgE in heparin anticoagulated plasma. Briefly, ELISA plates were coated with 100 μL of OVA (10 μg/mL) in coating buffer overnight at 4 °C. Nonspecific binding was blocked by incubating plates with 200 μL of 10% fetal calf serum in 0.1 M PBS for two hours at room temperature. Then 100 μL of mouse serum samples diluted in blocking buffer were added and incubated overnight at 4 °C. Following that 100 μL of biotinylated anti-IgA/IgG1/IgE detecting antibody (0.5 − 2.5 μg/mL in PBST) was added to the plate and incubated at room temperature for 90 min. Wells were then incubated with streptavidin-HRP conjugated following ABTS substrate to develop color. Plates were washed five times with PBST in between each step. A stop solution was added to reduce variability when reading the plate at OD 405 nm.

### Histopathological and pulmonary vascular permeability analysis

Mouse lungs were processed as described [29]. The right bronchus was ligated with a thread before the intratracheal instillation of 1 mL of cold 4% paraformaldehyde into left lung in situ. After the left lung was inflated, the right lung was cut and stored at − 80 °C for further analysis. The left lung was immersion fixed in 4% paraformaldehyde, processed, and embedded in paraffin in three pieces. The sections taken from all three pieces (5 µm thickness) were placed on poly-l-lysine coated glass slides and stained with hematoxylin and eosin for histopathological examination. Histopathology scoring was adopted from the previous description [40]. Briefly, the thickness of the bronchiolar and blood vessel walls, which we used to represent the thickness of the smooth muscle layer, was determined as the average distance between the inner edge to the outer edge of the wall at four different places on each sample. The inflammatory cells infiltrating out of the blood vessels and goblet cells along the bronchiolar epithelium were quantified. All lung sections were evaluated at 1000 × magnification and scored using a 4-point scale, as follows: 0, normal lung architecture; 1, minimal, a diffuse reaction in alveolar walls, congestion, 1 − 10 immune-cells/field in peribronchiolar vascular space; 2, mild, 11 − 20 immune-cells/field, congestion, slightly thickened bronchiolar and blood vessel walls, some goblet cells along the bronchiolar epithelium with their mucus product; 3, moderate, 21 − 30 immune-cells /field, congestion, thickened bronchiolar and blood vessel walls, light epithelial damage, moderate goblet cell hyperplasia; and 4, severe, ≥ 31 immune-cells /field, congestion, very thickened bronchiolar and blood vessel walls, severe goblet cell hyperplasia with a lot of their mucus product, more than 10% of lung consolidated, epithelial damage.

To evaluate vascular permeability, we performed protein analysis on BAL fluids by Bradford protein assay [41] using a protein assay kit (Bio-Rad, Hercule, CA.) following the manufacturer’s instructions.

### Immuno-gold electron microscopy for LSP1

After in situ intra-tracheal fixation, a piece of left mouse lung (1 × 2 mm^2^) was cut and fixed in 2% paraformaldehyde with 0.1% glutaraldehyde in 0.1 M sodium cacodylate buffer overnight at 4 °C. Next, the samples were rinsed in three changes of 0.1 M sodium cacodylate buffer at 4 °C. After being dehydrated in ethanol, the tissues were infiltrated in fresh white resin three times before being placed next to a Sylvania Blacklight Blue A448-5151T8/BLB in a cryostat at − 4 °C for polymerization. The tissues were then sectioned 100 nm thickness on nickel grids. Immuno-gold staining procedure with LSP1 antibody followed a protocol from our previous paper [29]. The tissues were imaged using a transmission electron microscope (Hitachi HT7700—XFlash 6T160, Germany) operated at 80 kV.

### Lung Myeloperoxidase (MPO) and Eosinophil Peroxidase Assay (EPO) quantification

MPO and EPO assay protocols were adapted from a previous protocol [29, 42]. Briefly, mouse lung samples were homogenized in 500 µl of 50 mM HEPES (Invitrogen, Burlington, ON, Canada) and then re-homogenized in 500 µl of 0.5% cetyltrimethyl ammonium chloride solution. Diluted MPO standards from human leukocytes (Sigma-Aldrich, St. Louis, MO, USA) and mouse lung samples were added to 96-well plate. The MPO substrate (3, 3’, 5, 5’-tetramethylbenzidine) was then added, followed by use of 1 M H2SO4 to terminate the reaction. The plate was read at 450 nm OD using NOVOstar software (Bio-Rad). Total protein concentrations in each sample was quantified using a protein assay kit (Bio-Rad). The data are expressed as units of MPO per mg of lung protein.

To assess EPO levels, samples and EPO standards were added to 96-well plates. Stop solution was added after two-minute incubation with eosinophil peroxidase assay substrate solution (3 mM O-phenylenediamine). EPO levels were read at 490 nm OD using NOVOstar software (Bio-Rad). Total protein concentration in each sample was quantified using a protein assay kit (Bio-Rad). Data were expressed as the units of EPO per mg of lung protein.

### LSP1, Gr1 and MPO immunohistochemical and immunofluorescent staining

The immunohistochemical staining protocol for LSP1 and the immunofluorescent protocols for staining LSP1 and Gr1, or LSP1 and MPO were modified from our previous report [29]. Briefly, sections were de-paraffinized, and treated to quench endogenous peroxidase activity and then for antigen retrieval. The non-specific binding in the lung sections was blocked with 1% BSA, followed by incubation with primary and appropriate secondary antibodies. Tissues were stained with 20 µg/ml rabbit anti-mouse LSP1 polyclonal antibody (Novus Biological, Oakville, ON, Canada) followed by secondary polyclonal goat anti-rabbit immunoglobulins/horse radish peroxidase (HRP). In immunofluorescent staining for LSP1 and MPO, tissues were stained with 20 µg/ml rabbit anti-mouse LSP1 polyclonal antibody (Novus Biological, Oakville, ON, Canada) and 20 µg/ml purified polyclonal goat anti-human/mouse myeloperoxidase antibody (R&D Systems, Minneapolis, MN, USA.) followed by 1:100 polyclonal goat anti-rabbit immunoglobulins IgG /conjugated Cy5 (Abcam, Toronto, ON, Canada) and 1:200 polyclonal donkey anti-goat immunoglobulins/conjugated AF488 (Life technology, Waltham, MA., USA.), respectively. In LSP1 and Gr1 immunofluorescent staining, tissues were stained with 20 µg/ml rabbit anti-mouse LSP1 polyclonal antibody, reactive in mouse and human (Novus Biological, Oakville, ON, Canada), and purified rat anti-mouse Gr1 antibody (Ly-6G and Ly-6C) (BD Biosciences Pharminogen™, Mississauga, ON, Canada), followed by goat anti-rabbit IgG secondary antibody conjugated Alexa fluor 488 (Life technology, Waltham, MA., USA.), and chicken anti-rat IgG secondary antibody conjugated Alexa fluor 647 (Life technology, Waltham, MA., USA.), respectively.

We have used LSP-1 antibody in our previous studies and have even performed pre-absorption controls [29]. The negative controls included staining with isotype antibody matching control rabbit IgG (Novus Biological, Oakville, ON, Canada), rat IgG2bκ and goat IgG isotype control antibodies (Santa Cruz Biotechnology, Mississauga, ON, Canada) instead of primary antibody for LSP1, Gr1 and MPO, respectively. Another negative control was the omission of primary antibody. Also, Lsp1^−/−^ murine lungs, stained with LSP1 antibody, acted as an important negative control for the non-specific staining by the antibody (Additional file 1: Fig. S1). Tissues were incubated for 5 min in 0.33 µg/ml DAPI in immunofluorescent staining or methyl green in immunohistochemical staining for staining the DNA of nuclei. For immunohistochemical staining, the color was developed by Vector® VIP peroxidase substrate kit for peroxidase (Vector Laboratories, Burlingame, CA, USA). For immunofluorescent staining, samples were imaged using a confocal scanning laser microscope (Leica TCS SP5 LSCM, Ontario, Canada) with a 63 × oil immersion objective lens.

### LSP1, Gr1 and MPO immunohistochemical and immunofluorescent staining on human lungs

Normal and asthmatic human lungs in paraffin were obtained from the Department of Pathology in the College of Medicine at the University of Saskatchewan (n = 3 each group). Human lung sections were stained with rabbit anti-mouse LSP1 antibody (Novus Biological, Oakville, ON, Canada) followed by polyclonal goat anti-rabbit immunoglobulins IgG conjugated Cy5 secondary antibody (1:100, Abcam, Toronto, ON, Canada). Human lung sections stained with bovine serum albumin or IgG rabbit isotype control instead of LSP1 primary antibody served as negative controls. Samples were imaged using Olympus IX83 inverted microscope with a total internal reflection fluorescence (TIRF) system under a 10 × , and 60 × oil immersion objective lens. The controls are shown in Additional file 1: Fig. S2.

### Western blot analyses for LSP1

Frozen mouse lungs were homogenized in T-PER Tissue Protein Extraction Reagent (Thermo Scientific, Rockford, IL., USA) with protease and phosphatase inhibitor cocktails as described [43]. Total protein concentration in each sample was quantified using a protein assay kit (Bio-Rad). The Western blot procedure has been reported previously [29, 44]. Densitometry quantification was performed using ImageJ software (from the National Institutes of Health and the Laboratory for Optical and Computational Instrumentation, University of Wisconsin) to evaluate relative density of total LSP1 expression levels adjusted to beta-actin. Full blots are included in Additional file 1: Fig. S2.

### Statistical analysis

Statistical analysis was performed using GraphPad Prism software version 5.04 (San Diego, CA, USA). Quantitative results were expressed as mean ± SEM. The normal distribution of residuals was tested by histogram and Shapiro–Wilk test. Data were analyzed by ANOVA, followed by Bonferroni multiple comparison test. Student t-test or Wilcoxon Signed Rank Test was used to compare two groups. The critical value of α was set to 0.05 as a significant difference (two-tailed).

## Results

### LSP1 expression was increased in OVA-induced murine asthma

**Fig. 1:**
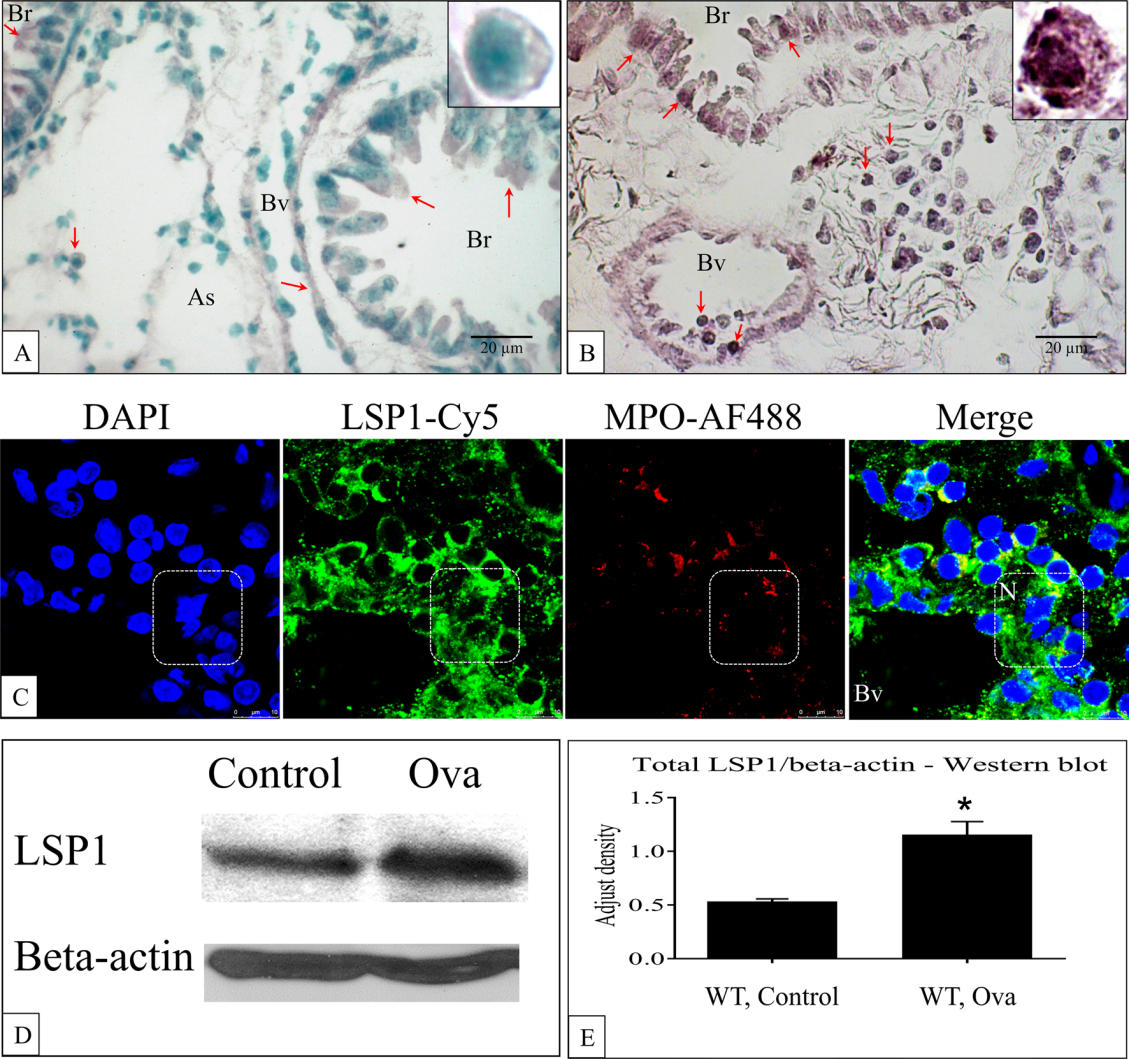
The increased expression of LSP1 in asthma mouse lungs. LSP-1 staining is observed in lung sections from control mouse **A** and OVA-challenged mouse lungs (**B**). The staining is observed in bronchiolar epithelium, and endothelium (arrows). High magnification insets in **A** and **B** show staining in alveolar macrophages (Magnification: 400 × and 1000 ×). **C** The representative confocal images of OVA mouse lung sections show staining for LSP-1 (green) and myeloperoxidase (MPO; red). The merged image show LSP-1 to be predominantly in neutrophils. Western blots **D** and the densitometry **E** for LSP1 (at about 52 kD) and β-actin (47 kD) showed that OVA mouse lungs have higher LSP1 levels than control mouse lungs. Data were expressed as mean ± SEM. Asterisk (*) indicates significant difference from wildtype control (P < 0.05, n = 3 each group). *As* alveolar space, *Bv* blood vessel, *Br* bronchiole, *N* Neutrophil, *WT* wildtype

Immunohistochemistry confirmed the expression of LSP1 in the macrophages, bronchiolar epithelium, airway epithelium, and vascular endothelium in normal healthy mouse lungs of WT mice (Fig. 1A). The LSP1 staining was more intense in these cells in the lungs of OVA challenged mice (Fig. 1B). The controls for the immunohistochemistry are included in Additional file 1: Fig. S1. Dual immunostaining for LSP-1 and MPO, as a marker for neutrophils [45] (although it may also be expressed in some monocytes) showed strong LSP1 expression in the plasma membrane and cytoplasm with weaker staining in the nuclei of neutrophils (Fig. 1C). Western blot data demonstrated higher levels of total LSP1 in the lungs of OVA-challenged WT mice compared to control mice (P < 0.05, Fig. 1D, E Additional file 1: Fig. S2).

**Fig. 2:**
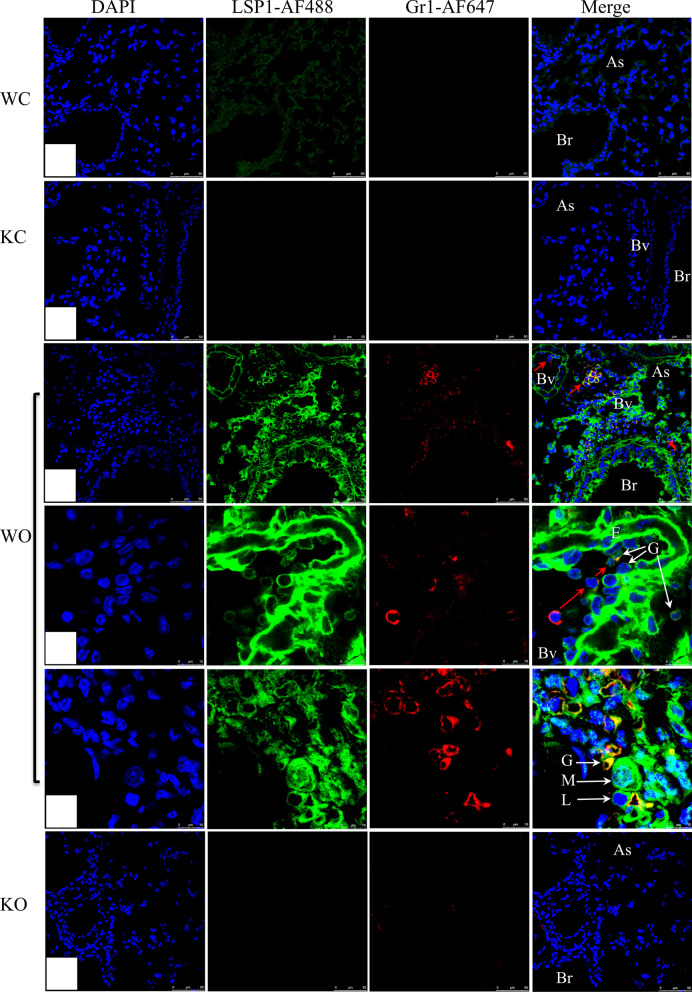
Immunofluorescent staining LSP1 and granulocytes in mouse lungs. Mouse lung sections display a lack of LSP-1 staining (green) and granulocytes (red) in LSP-1^−/−^ control (KO) and OVA-challenged mice (KO) compared to the wild-type control (WC) and wild-type OVA (WO) mice. *As* alveolar space, *Bv* blood vessel, *Br* bronchiole, *E* endothelium, *G* granulocytes, *L* lymphocytes, *M* macrophages, *N* neutrophils. n = 3 each group

**Fig. 3:**
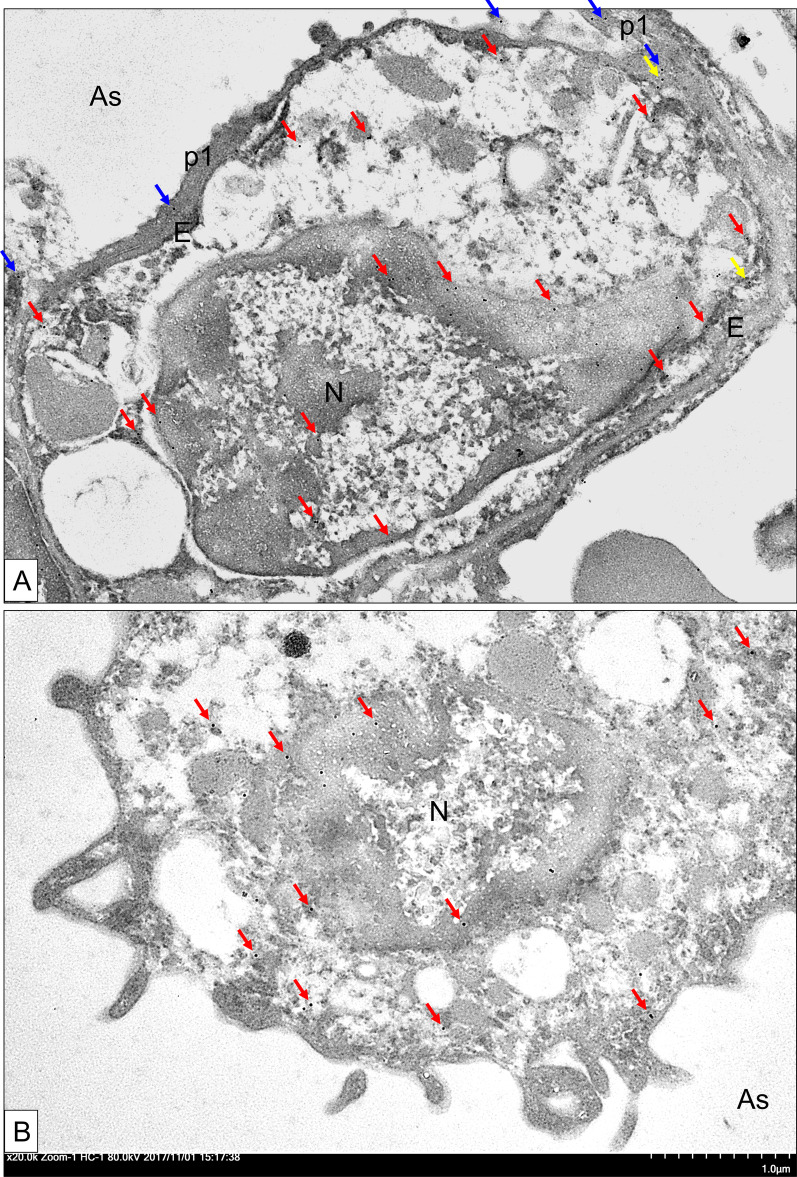
The immuno-gold electron micrographs for the expression of LSP1 in the mouse lung. The transmission electron micrograph of an OVA-induced asthmatic WT mouse lung showed LSP1 staining in the plasma membrane, nucleus (N) and cytoplasm of an intravascular macrophage **A** and alveolar macrophage (red arrows, **B**), an endothelial cell (E, yellow arrows, **A**), and type I pneumocytes (p1, blue arrows, **A**). Original magnification 20,000 ×

Dual immune-fluorescence revealed LSP1 expression in all Gr1-positive granulocytes, which would include neutrophils and eosinophils (Fig. 2). Interestingly, the LSP1 fluorescence intensity was stronger in Gr1 cells adhering to endothelium or alveoli than those cells in blood vessels (Fig. 2C, D). We also observed LPS1 staining in the macrophages and lymphocytes in OVA-challenged mouse lungs (Fig. 2E). The negative immunohistochemical controls showed no staining (Additional file 1: Fig. S3). Immuno-gold staining with LSP1 antibody further confirmed the expression of LSP1 on the plasma membrane, cytoplasm, and nucleus of pulmonary intravascular macrophages (Fig. 3A) and alveolar macrophages (Fig. 3B).

### LSP1 deficiency reduced the histopathologic signs of lung inflammation and AHR in mouse

**Fig. 4:**
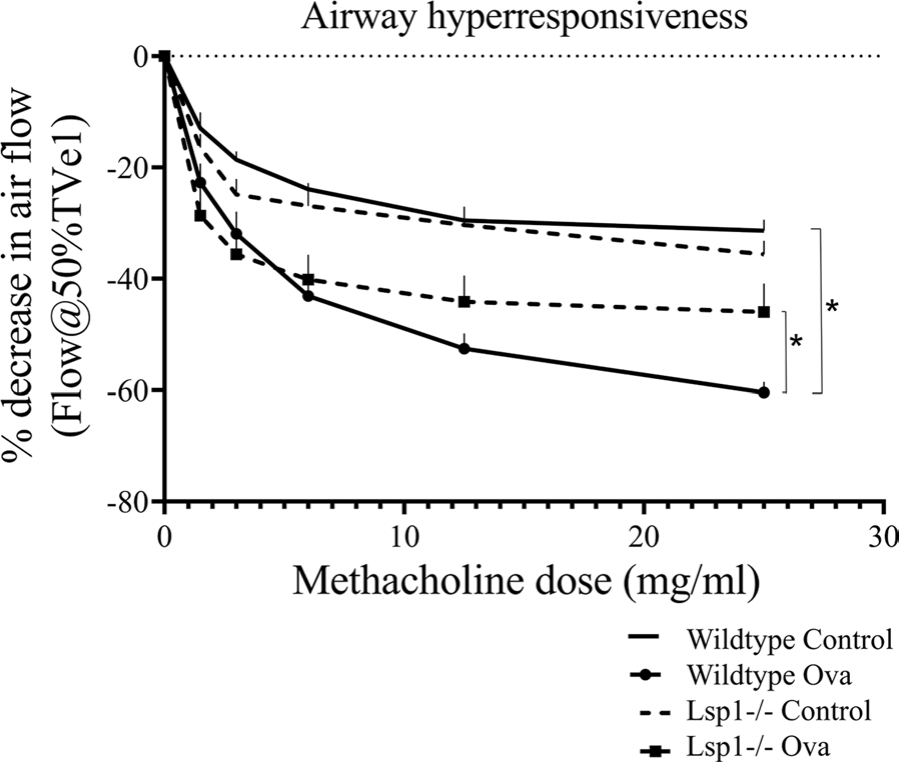
Knocking-out LSP1 ameliorated AHR in the OVA-induced asthma mouse model. Statistical linear regression shows that the slopes of the AHR curves for the WT OVA mice (Y = − 1.958*X–19.48) and Lsp1^−/−^ OVA mice (Y = − 1.206*X–22.79) are significant different (P < 0.05, n = 6 each group). It suggested that the AHR of Lsp1^−/−^ OVA mice was improved compared with that of WT OVA mice. Data expressed as mean ± SEM at each methacholine dose

The data showed that control WT and Lsp1^−/−^ mice responded similarly when exposed to increasing doses of methacholine. However, when compared to WT OVA mice, Lsp1^−/−^ OVA mice showed significantly more decline in AHR when exposed to 25 mg/mL methacholine aerosols. The statistical linear regression further showed significant differences in the slopes of WT OVA mice (Y = -1.958*X—19.48) and Lsp1^−/−^ OVA mice (Y = − 1.206*X—22.79) (Fig. 4).

**Fig. 5:**
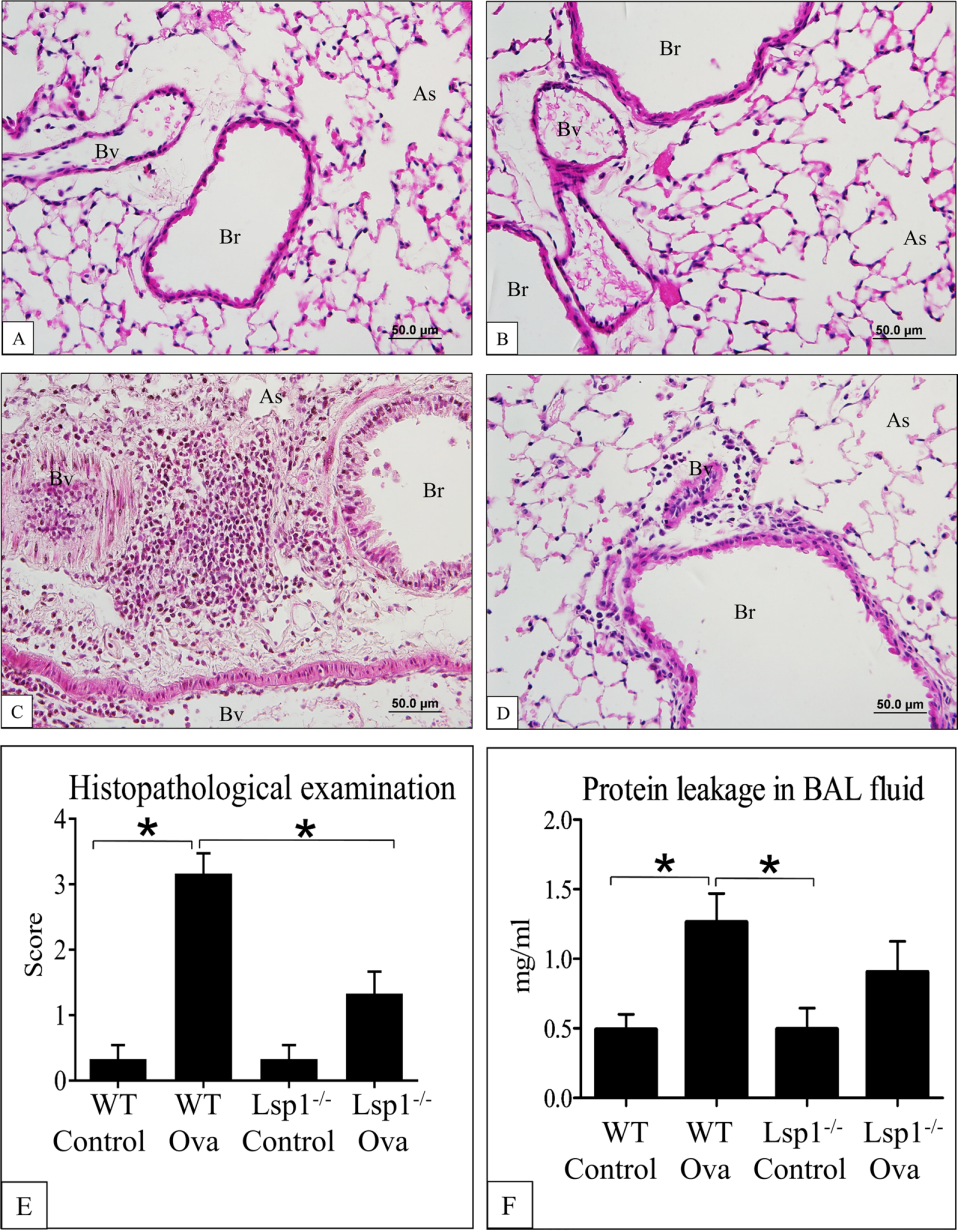
The histopathological examination of mouse lungs with hematoxylin and eosin staining. Control wildtype **A** and Lsp1^−/−^ mice **B** display normal appearing alveolar septa and alveoli. In comparison to Lsp1^−/−^ OVA lungs (**D**), WT OVA mouse lungs **C** showed more inflammatory cell infiltration in alveolar septa, alveoli, and peri-bronchial and peri-vascular spaces. **E** Semi-quantitative scoring showed more severe pathology in WT OVA and Lsp1^−/−^ OVA mice than in their respective control controls, and more in WT OVA mice, than in Lsp1^−/−^ OVA mice. **F** The a*nalysis of* protein concentration in BAL fluid showed that *b*oth types of mice challenged with OVA had significantly higher protein concentration than control but there was no significant difference between WT and Lsp1^−/−^ mice. *As* Alveolar space, *Bv* Blood vessel, *Br* Bronchiole, *PVS* Peribronchiolar vascular space, *WT* wildtype. Magnification: 400 × . Asterisk (*) indicates a significant difference (P < 0.05, n = 6 each group)

The normal lung sections from WT (Fig. 5A) and Lsp1^−/−^ mice (Fig. 5B) showed no inflammation and normal histology with thin alveolar septa, clear alveolar spaces, and occasional alveolar macrophages. Lungs from OVA-challenged WT mice (Fig. 5C) showed more lung inflammation compared to Lsp1^−/−^ OVA mouse lungs (Fig. 5D). The histological scoring as described in the methods revealed significantly more lung pathology in WT OVA mice compared to all other groups (Fig. 5E). The BAL fluid of both genotypes of mice challenged with OVA had a significantly higher level of protein concentration compared to respective normal controls. There was no difference between OVA-treated WT and Lsp1^−/−^ mice in protein concentration in BALF (Fig. 5F).

### LSP1 deficiency dramatically down-regulated inflammatory cells recruitment into inflamed lungs

**Fig. 6:**
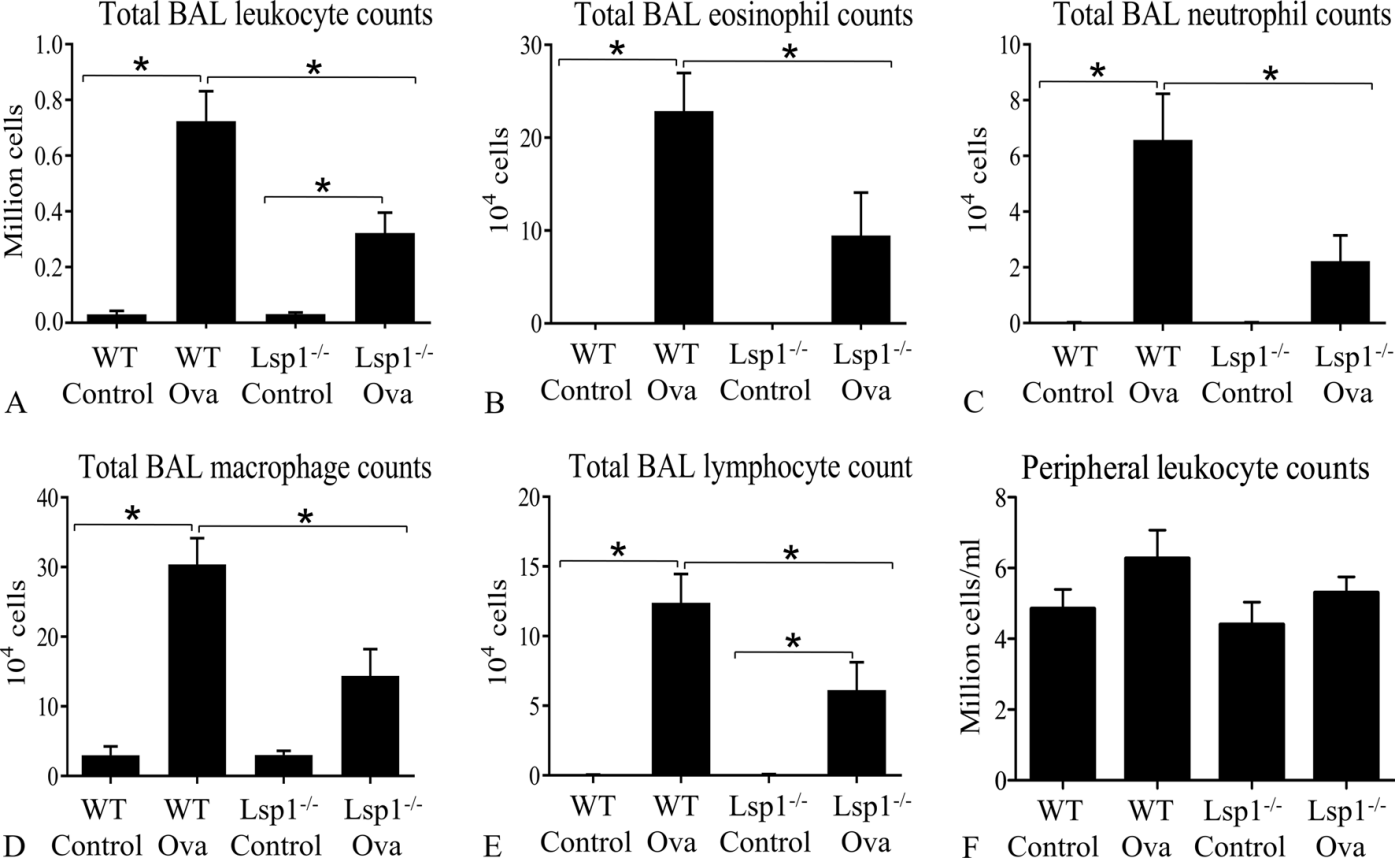
Total and differential leukocyte counts in bronchoalveolar lavage (BAL) fluid and peripheral blood. LSP1-knockout attenuates immunoinflammatory cell recruitment into the alveolar space in the OVA-induced asthma mouse model (**A**–**E**). There were not any statistically significant differences between any groups in terms of peripheral blood leukocyte numbers (**F**). Data were expressed as mean ± SEM. Asterisk (*) indicates a significant difference (P < 0.05, n = 6 each group)

**Fig. 7:**
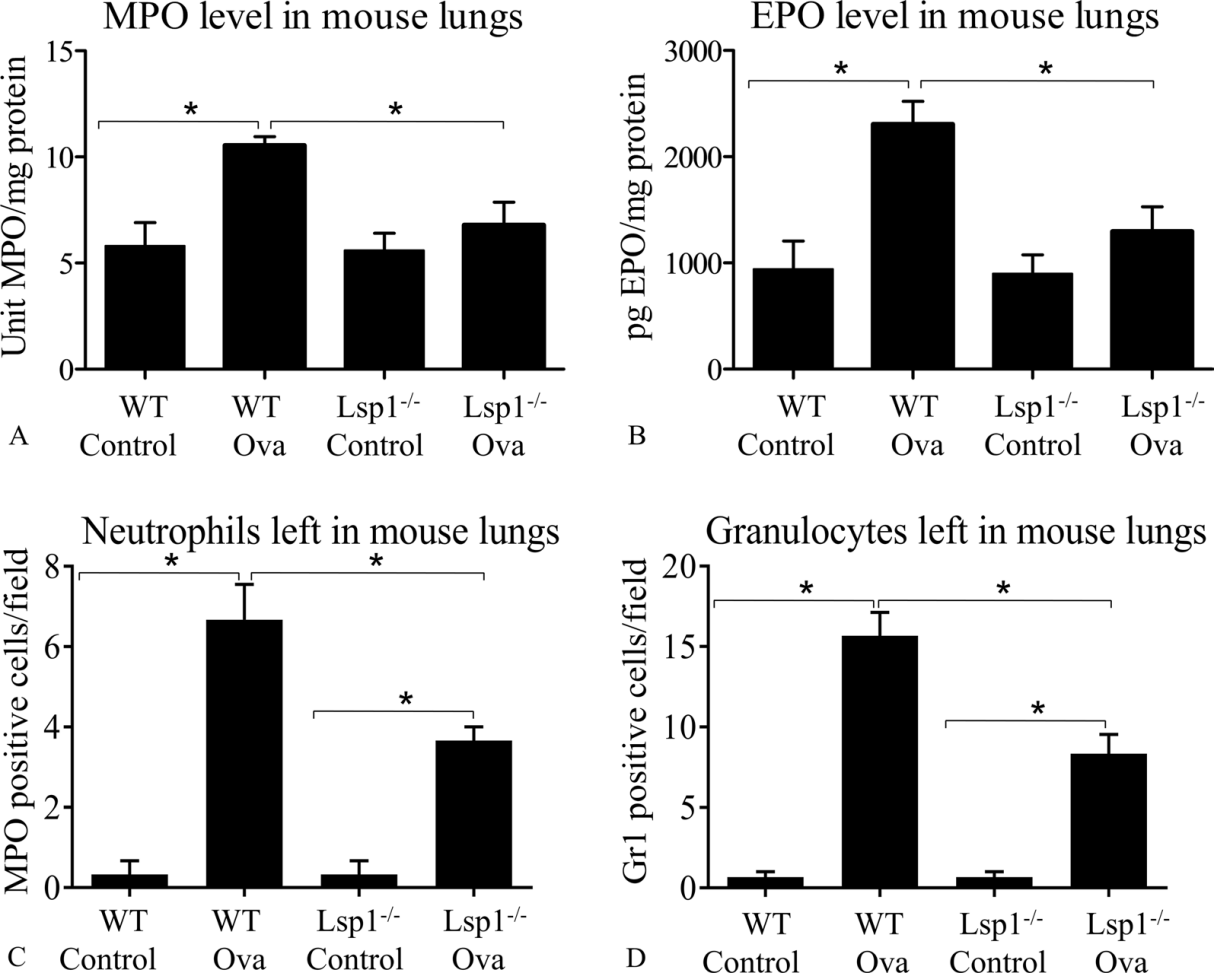
The quantification of neutrophils and eosinophils left in mouse lungs after bronchoalveolar lavage. LSP1 deficiency reduced asthma-associated neutrophil and eosinophil migration into the lungs. **A**, **B** Mouse lung lysates were analyzed using MPO and EPO assays as surrogate measures for the numbers of neutrophils and eosinophils, respectively, remaining in the lung after bronchoalveolar lavage (n = 6 each group). **C**, **D** We counted the number of neutrophils and granulocytes in MPO and Gr1 immunofluorescent-stained mouse lungs, respectively (n = 3 each group). Data were expressed as mean ± SEM. Asterisk (*) indicates a significant difference (P < 0.05)

Compared to OVA-challenged Lsp1^−/−^ mice, WT OVA mice had significantly more leukocytes, including eosinophils, neutrophils, macrophages, and lymphocytes in BAL fluid (P < 0.05, n = 6 each group) (Fig. 6A − 6E; Additional file 1: Fig. S3). There were however no differences in peripheral blood leukocyte numbers between control and OVA-challenged as well as between WT and Lsp1^−/−^ mice (Fig. 6F). The data also showed that OVA-challenged WT mice had higher levels of MPO and EPO compared to Lsp1^−/−^ OVA mice (P < 0.05, n = 6 each group) (Fig. 7A, B). The Gr1 antibody staining showed significantly higher numbers of neutrophils and eosinophils in the lungs of WT OVA mice compared to Lsp1^−/−^ OVA mouse lungs (P < 0.05, n = 3 each group) (Fig. 2, and Fig. 7D). Also, Lsp1^−/−^ OVA mice had a fewer MPO-labeled cells, likely neutrophils, recruited to the perivascular space than WT OVA mice (P < 0.05, n = 3 each group) (Fig. 7C).

### The absence of LSP1 gene significantly attenuated the levels of IL-4, IL-5, IL-6, IL-13, and CXCL1 in the BAL fluid as well as ovalbumin-specific IgE in the serum of OVA-challenged mice

**Fig. 8:**
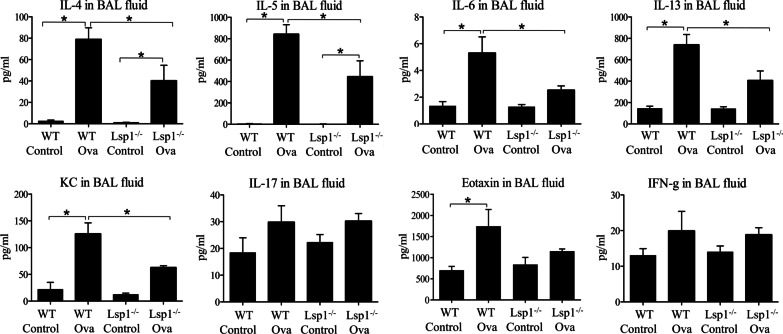
The quantification of cytokines and chemokines in BAL fluid using Bioplex assay. LSP1^−/−^ OVA mice had significantly lower BAL concentrations of IL-4, IL-5, IL-6, IL-13, and CXCL1, but not of IL-17, CCL11, or IFN-γ compared to wildtype OVA mice. Data were expressed as mean ± SEM. Asterisk (*) indicates a significant difference (P < 0.05, n = 6 each group). *IL* interleukin, *CXCL1* keratinocyte-derived chemokine, *IFN-γ* interferon γ

Bioplex assays to measure the concentrations of IL-4, IL-5, IL-6, IL-13, CXCL1, IL-17, CCL11, and IFN-γ in BAL showed no difference in IL-17, CCL11, and IFN-γ between WT or Lsp1^−/−^ mice treated with OVA. However, the concentrations of IL-4, IL-5, IL-6, IL-13, and CXCL1 were increased in the BAL of WT OVA mice compared to WT control mice as well as Lsp1^−/−^ OVA mice (P < 0.05, n = 6 each group, Fig. 8).

**Fig. 9:**
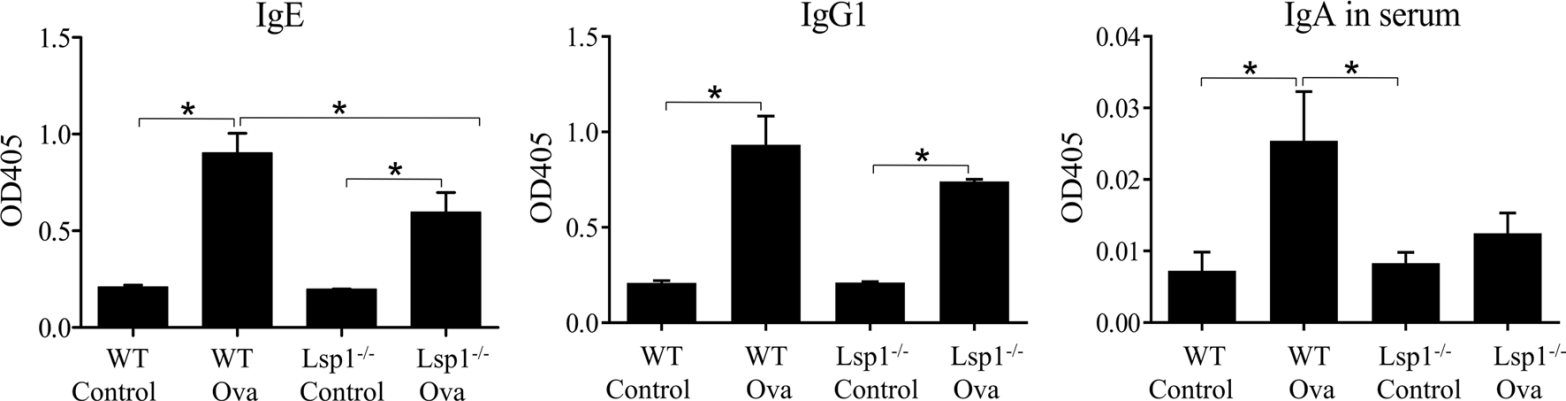
The impact of LSP1 expression on OVA-specific IgE, IgG1, and IgA levels in serum of asthmatic mice. Wildtype OVA mice had statistically significant increases inOVA-specific IgE, IgG1, IgA levels compared with WT control mice. The levels of OVA-specific IgE found in WT OVA mice was significantly higher than that in Lsp1^−/−^ OVA mice. Data were expressed as mean ± SEM. Asterisk (*) indicates significant difference (P < 0.05, n = 6 each group)

ELISA data showed higher concentrations of ovalbumin-specific IgE and IgG1 in serum of both WT and Lsp1^−/−^ OVA-challenged mice compared to control mice (P < 0.05, n = 6 each group) (Fig. 9). The ovalbumin-specific IgE but not IgA concentration in serum of OVA-challenged WT mice was higher than that of Lsp1^−/−^ OVA mice (P < 0.05, n = 6 each group; Fig. 9).

**Fig. 10:**
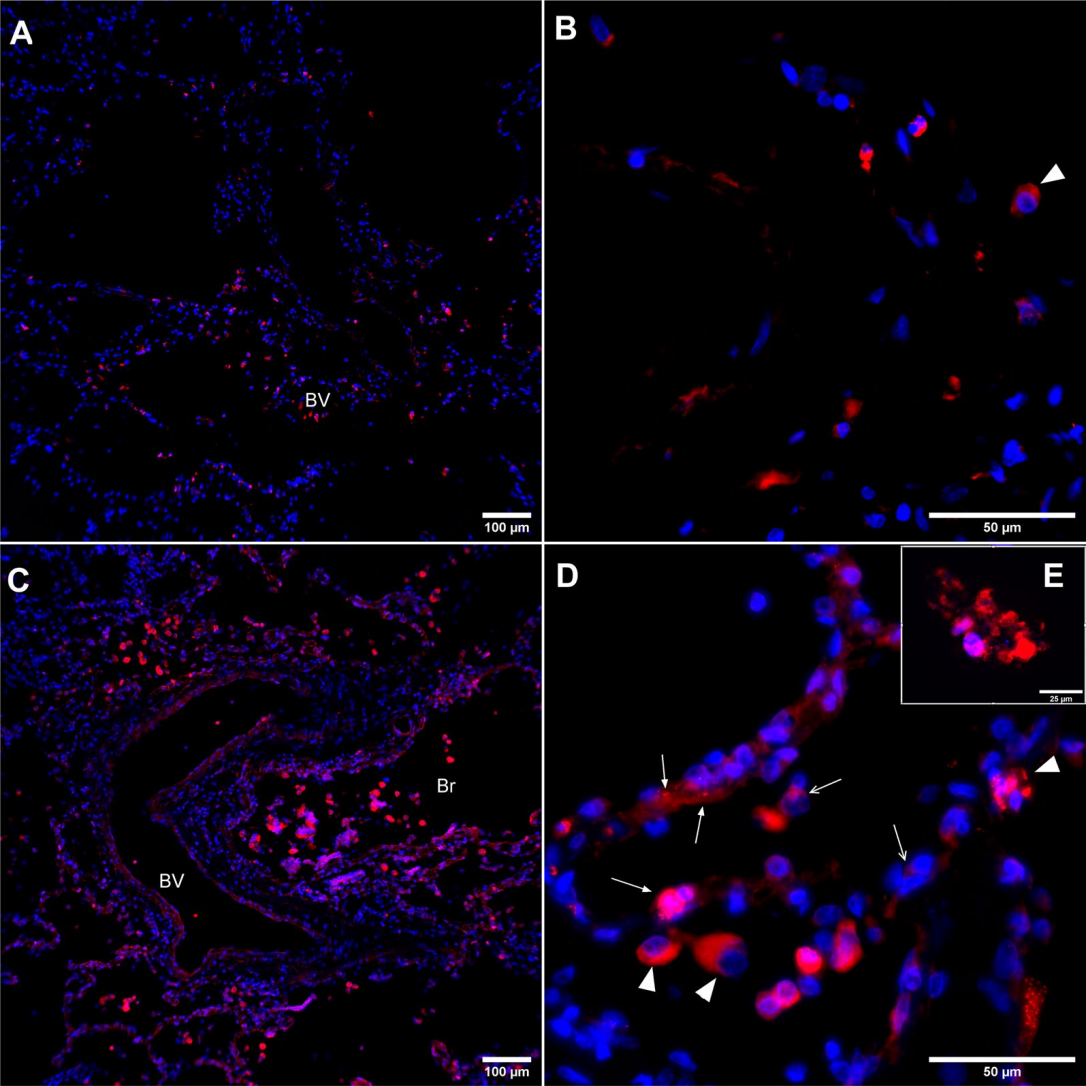
LSP1 expression in normal and asthmatic human lungs. In the normal human lungs (**A**, **B**), LSP1 (red) is seen in the alveolar septa and blood vessels (BV) and in macrophages (arrowheads). The expression is more robust in the asthmatic lung **C**, **D** and **E** in blood vessels (BV) and bronchioles (Br), which also show many more inflammatory cells. Alveolar macrophages (arrowheads) and granulocytes (arrows) show staining for LSP1. (n = 3 each group)

Finally, normal human lungs were reactive for LSP1 in the endothelium, macrophages and the alveolar septa ( Fig. 10A, B). Asthmatic lung tissues showed large numbers of inflammatory cells in alveolar and perivascular spaces. These inflammatory cells including macrophages and neutrophils were intensely positive for LSP1 in their cytoplasm and the plasma membrane (Fig. 10C–E).

## Discussion

We provide new data on the role of LSP-1 in regulation of AHR and lung inflammation in a mouse model of asthma. The data show deficiency of LSP-1 reduces AHR and lung inflammation. In addition, we also reported increases in the expression of LSP1 in various resident and recruited cells in asthmatic lungs from the mice and humans. These data build on our previous report [29] and further establishes the role of LPS1 as an important regulator of inflammation in the lungs.

The OVA-induced murine model of asthma is an important tool in elucidating the mechanisms of acute asthma in humans such as recruitment of inflammatory cells and AHR [46–48]. The recruitment of inflammatory cells such as eosinophils, neutrophils and lymphocytes into the lungs is an important feature of asthma [49] and we observed the same in our model based on BAL analyses, immunohistology and MPO assays. The deficiency of LSP1 led to significant reduction in the recruitment of neutrophils, eosinophils, lymphocytes and macrophages into the airways of OVA-treated mice compared to their WT counterparts. These data align with our previous findings of LSP1 deficiency being associated with decreased inflammatory cell recruitment in an endotoxin-induced lung inflammation study [29]. LSP1 has been shown to regulate T-lymphocyte migration in rheumatoid arthritis [31]. Our finding of significant reduction in lymphocyte numbers in lungs of LSP1 deficient OVA-challenged mice may be of significance considering there are earlier studies linking the alveolar migration of T-lymphocytes in asthma [50, 51]. It has been previously reported that the increasing numbers of eosinophils, mast cells and neutrophils along with their enzymic products cause damage to lung tissues in and determine severity of asthma [52–54]. The recurring episodes of eosinophilia and pulmonary migration of eosinophils in asthmatics lead to thickening of the sub-epithelial basement membrane, bronchial hyperresponsiveness, and epithelial damage [53, 54]. Neutrophil production of mediators such as elastase, or neutrophil interactions with goblet cells leading to mucus accumulation can narrow the airway. Degranulation of goblet cells depends on interactions with migrated neutrophils, and specifically their elastase activity and the expression of the adhesive molecules such as intercellular adhesion molecule-1 (ICAM-1), CD18, and CD11b in vivo [55]. Therefore, reduced recruitment of inflammatory cells observed in Lsp1^−/−^ may lead to better physiological outcomes in asthma. Previous data has indicated that lack of LSP1 may not affect MAPK cell signaling, but phosphorylation of LPS1 itself may lead to modulation of the actin cytoskeleton of neutrophils, facilitating their migration [29, 56, 57].

The inflammatory mediators in asthma have been extensively studied for their roles in cell recruitment and cell activation [20, 21, 58]. We also quantified a number of cytokines in the BAL from the mice and found that WT OVA mice had significantly higher concentrations of IL-4, IL-5, IL-6, IL-13, and CXCL1 compared to the Lsp1^−/−^ mice. There could be multiple reasons for this observation. The reduced migration of inflammatory cells, which are major sources of cytokines, in OVA-challenged Lsp1^−/−^ mice would have contributed to the lower levels of selected cytokines. The absence of LSP1 expression, which can act as a substrate for MAPK, [56] in lymphocytes may attenuate cytokine production by T lymphocytes (IL-4, IL-5, IL-13), leading to reduced eosinophil recruitment and IgE production by B plasma cell. It is well-known that IL-4, IL-5, and IL-13 cytokines produced by CD4^+^ natural killer T cells and CD4^+^ T MHCII-restricted cells enhance eosinophilia and increase the severity of asthma [59, 60]. IL-13 stimulates the epithelium in the airways [61]. The airway epithelium also produces IL-5, IL-2, TGF-β, IL-6 and IL-10, which promote B cell differentiation into plasma cells to produce IgA [62, 63]. These cytokines also cause the metaplasia of goblet cells and alterations in epithelial-mesenchymal signaling resulting in sub-epithelial fibrosis or smooth muscle hyperplasia [64]. Eosinophils, which were reduced in numbers in Lsp1^−/−^ asthmatic mice, produce IL-16 to attract CD4^+^ T cell in asthma [65]. Previous studies have shown that binding of IgA, IgG, and IgE to their receptors on eosinophils activates and degranulates them [62, 63]. We found higher levels of OVA-specific IgG but not IgA in the serum of WT asthmatic mice compared to Lsp1^−/−^ OVA mice. Taken together, we believe that LSP1 deficiency disrupts recruitment of inflammatory cells and production of cytokines and thereby alleviates physiological and inflammatory outcomes in our murine model of asthma.

The AHR to methacholine is a reliable method to evaluate airway function and has been shown to correlate well with the invasive methods that are considered gold standard for measuring lung mechanics [66] [67]. Along with diminished recruitment of inflammatory cells and expression of certain cytokines, there was reduction in AHR, one of our major physiological measurements, in Lsp1^−/−^ asthmatic mice. Our experiments don’t directly address the underlying mechanisms through which LSP-1 influences AHR, but the association between AHR and inflammation has also not yet been fully resolved [68]. There however are data to show that the asthma-related AHR is an outcome of excessive broncho-constriction due to hypertrophy or hyperplasia of bronchiolar smooth muscles with repeated episodes of eosinophil-related airway inflammation [68, 69] [70] [71]. We believe that reduced lung inflammation in Lsp1^−/−^ mice may have led to improvement in airflow into the lungs by reducing AHR.

Lastly, to set the stage for the next set of experiments focused on human cells and tissues, we evaluated expression of LSP-1 in normal and asthmatic human lungs. The increase in LSP1 expression on various resident and recruited cells in the asthmatic lungs alludes to a potential for this protein in the pathogenesis of asthma. These data are similar to the increase in LSP1 staining in lung samples from sepsis patients [29]. The next set of studies will focus on the role of LSP1 in regulating the function of human immune cells.

## Conclusions

The study provides new data that deficiency of LSP-1 reduces lung inflammation as well as AHR in a murine model of OVA-induced asthma. LSP-1 deficiency likely disrupts the fundamental inflammatory process of recruitment of neutrophils and eosinophils and the associated network of cytokines to reduce inflammation and the physiological outcome of increased AHR. These data are in line with the role of LSP-1 in inflammatory cell recruitment in endotoxin-induced lung inflammation.

## Supplementary Information

**Additional file 1: Figure S1.** LSP1 immunohistochemical staining controls for Fig. 2A, B. **Figure S2.** LSP1, MPO, and Gr1 immunofluorescent staining controls for Fig. 2C and Fig. 3. **Figure S3.** Cytospun BAL cells were stained with Hemacolor stain kit

## Data Availability

The datasets used and/or analysed during the current study are available from the corresponding author on reasonable request.
