# Supplementary material for: Deficiency of leukocyte-specific protein 1 (LSP1) alleviates asthmatic inflammation in a mouse model
Source: Respir Res. 2022 Jun 22;23:165. doi: 10.1186/s12931-022-02078-7 (PMC9219131; doi:10.1186/s12931-022-02078-7)
Supplement: Supplementary file 1 — Additional file 1: Figure S1. LSP1 immunohistochemical staining controls for Fig. 2A, B. Figure S2. LSP1, MPO, and Gr1 immunofluorescent staining controls for Fig. 2C and Fig. 3. Figure S3. Cytospun BAL cells were stained with Hemacolor stain kit [file 12931_2022_2078_MOESM1_ESM.docx]

**Additional file 1: Figure S1. LSP1 immunohistochemical staining controls for Figure 2A and 2B.** (A) WT mouse lung sections, stained with vWF antibody as a positive control for the protocol, reacted only with vascular endothelium (arrow, pink-violet). Mouse lung sections, omitting LSP1 primary antibody (B) or staining with IgG rabbit isotype control (C) instead of LSP1 primary antibody, showed only the green-blue color of methyl green counterstaining. *Lsp1^−/−^* mouse lung sections, stained with LSP1 antibody (D), did not show any positive reaction with the antibody. Magnification: 400×.

**Additional file 1: Figure S2. LSP1, MPO, and Gr1 immunofluorescent staining controls for figure 2C and figure 3.** (A) Mouse lung sections were stained with IgG rabbit isotype control instead of LSP1 primary antibody and IgG goat isotype control instead of MPO primary antibody. (B) Mouse lung sections, omitting LSP1 and MPO primary antibodies, were stained with secondary antibody anti-rabbit IgG conjugated Cy5, secondary antibody anti-goat IgG conjugated Alexa Fluor 488. (C) Mouse lung sections were stained with IgG rabbit isotype control instead of LSP1 primary antibody and IgG2bk rat isotype control instead of the Gr1 primary antibody. (D) Mouse lung sections, omitting LSP1 and Gr1 primary antibody, were stained with goat anti-rabbit IgG secondary antibody conjugated Alexa fluor 488, chicken anti-rat IgG secondary antibody conjugated Alexa fluor 647. All negative controls did not show any positive staining. Samples were imaged using a confocal scanning laser microscope Leica TCS SP5 LSCM with a 63× objective lens under oil immersion.

**Additional file 1: Figure S3. Cytospun BAL cells were stained with Hemacolor stain kit.** BAL cell population from the lungs of WT normal (Figure A) and LSP1 knock-out normal mice (Figure C) have only macrophages. BAL cell population from the lungs of wildtype asthma (Figure B) and LSP1 knock-out asthma (Figure D) have eosinophils (E), lymphocytes (L), macrophages (M), and neutrophils (N).
